# Novel thermophilic genera *Geochorda* gen. nov. and *Carboxydochorda* gen. nov. from the deep terrestrial subsurface reveal the ecophysiological diversity in the class *Limnochordia*

**DOI:** 10.3389/fmicb.2024.1441865

**Published:** 2024-09-23

**Authors:** Olga V. Karnachuk, Anastasia P. Lukina, Marat R. Avakyan, Vitaly V. Kadnikov, Shahjahon Begmatov, Alexey V. Beletsky, Ksenia G. Vlasova, Andrei A. Novikov, Viktoria A. Shcherbakova, Andrey V. Mardanov, Nikolai V. Ravin

**Affiliations:** ^1^Laboratory of Biochemistry and Molecular Biology, Tomsk State University, Tomsk, Russia; ^2^Institute of Bioengineering, Research Centre of Biotechnology of the Russian Academy of Sciences, Moscow, Russia; ^3^Gubkin University, Moscow, Russia; ^4^Skryabin Institute of Biochemistry and Physiology of Microorganisms, Federal Research Center Pushchino Center for Biological Research of the Russian Academy of Sciences, Moscow, Russia

**Keywords:** deep terrestrial subsurface, *Limnochordia*, thermophiles, carboxydotrophs, aerobic respiration, didermic cell wall

## Abstract

The class *Limnochordia* harbors a single cultivated member, the mesophilic *Limnochorda pilosa*, which was isolated from a meromictic lake. Despite numerous molecular signatures reported in various ecosystems, the ecophysiological versatility of this deeply branched lineage of *Firmicutes (Bacillota)* remains poorly understood. The objective of this study was to use targeted cultivation, based on metagenome-assembled genomes from a deep terrestrial aquifer in Western Siberia, to isolate two new thermophilic members of the class. These isolates, described as *Geochorda subterranea* gen. nov. sp. nov. and *Carboxydochorda subterranea* gen. nov. sp. nov. within the *Geochordaceae* fam. nov., were capable of both anaerobic and aerobic respiration using fumarate and O_2_, respectively, with simple sugars as electron donors. The cultivated *Geochordaceae* have demonstrated fermentative growth and degradation of various polymers, including starch, maltose, maltodextrin, xylan, and chitin. The carboxydotrophic *C. subterranea* sp. nov. exhibited autotrophic growth via the Calvin–Benson–Bassham cycle, using CO, H_2_, and formate as electron donors and O_2_ as an electron acceptor, adding metabolic flexibility to the bacterium in the nutrient-depleted “deep biosphere” and supporting the possibility of aerobic metabolism in the deep subsurface. The broad physiological potential deciphered from physiological experiments and comparative genomic data explains the widespread distribution of uncultivated members of the class *Limnochordia* in various ecosystems, where they can oxidize complex organic substrates through both aerobic and anaerobic respiration, as well as pursue a chemolithotrophic lifestyle through the oxidation of H_2_ or CO.

## 1 Introduction

It is widely accepted that cultivated bacteria and archaea represent only a small portion of the uncultivated majority of the prokaryotic world, known to us through environmental DNA sequences. The cultivation of microorganisms is not only necessary to validate novel taxa but also provides the basis for deciphering a growing number of microbial proteins with unknown functions. Careful consideration of metabolic pathways in metagenome-assembled genomes (MAGs) from uncultivated prokaryotes provides essential clues to their cultivation. The class *Limnochordia*, representing a distant phylogenetic lineage in the phylum *Firmicutes* (recently renamed *Bacillota*), is an example of a higher-ranking bacterial taxon with only one cultivated species. This class was created to accommodate the mesophilic *Limnochorda pilosa* HC45^T^, isolated from meromictic lake sediments in Japan (Watanabe et al., 2015). The isolate was distantly related to the genera *Simbiobacterium, Thermaerobacter*, and *Sulfobacillus* and had a number of specific characteristics that distinguish the strain from the canonical *Firmicites*, including a high DNA G+C content, a high percentage of GTC start codons, and the absence of several conserved genes involved in cell division (Watanabe et al., 2016). Later, metagenomic analysis revealed *Limnochordia* in various surface biotopes, including thermophilic compost (Braga et al., 2021), cattle manure enrichment hydrolyzing carboxymethyl cellulose (Scheffer et al., 2021), aged refuse soil (Hou et al., 2021), pit mud used for the production of Chinese beverage Baijiu (Shoubao et al., 2023), and mice cadaver brain immersed in fresh water (Wang et al., 2022). *Limnochordia* was also one of the main components of the microbial consortium applied for lignocellulose degradation (Liu et al., 2021).

A recent phylogenetic analysis of uncultivated *Firmicutes* from the uncultured bacteria and archaea (UBA) dataset of Parks et al. (2017) placed *Limnochordia* as the second-deepest-branching lineage after the *Halanaerobiales* (Taib et al., 2020). One unique characteristic of the *Limnochordia* is the presence of an outer membrane and a diderm (Gram-negative) character of the cell envelope. Phylogenomic analysis of the outer membrane markers in *Limnochordia, Negativicutes*, and *Halanaerobiales* has led the authors to hypothesize a two-membrane cell envelope in the ancestor of all *Firmicutes* and the multiple loss of the outer membrane during the diversification of the phylum (Taib et al., 2020). A recent rooted bacterial phylogeny has confirmed the diderm character of the last bacterial common ancestor (LBCA) (Coleman et al., 2021). Most of the 46 unclassified UBA genomes of uncultivated *Limnochordia* used for phylogenomic analysis by Taib et al. (2020) were assembled from anaerobic mud and digester samples. Until now, *L. pilosa* remained the only cultivated species in the class *Limnochordia*, and the ecophysiological versatility of this group remains largely understudied due to the lack of cultivated representatives.

A characteristic feature of deep terrestrial subsurface biotopes is the scarcity of energy sources for microbial life. There is a general consensus that deep subsurface environments are nutrient-depleted, oligotrophic, and energy-constrained (Purkamo et al., 2017; Westmeijer et al., 2022). The absence of light precludes photosynthetic production. The chemosynthetic production of organic carbon in the deep subsurface is highly variable due to the lack of a clear, constant supply of inorganic electron donors, including CH_4_, HS^−^, or Fe(II), as occurs in marine hydrothermal vents. Dihydrogen is the most common electron donor in the deep terrestrial biosphere (Ruff et al., 2023). Serpentinization produces H_2_ as a result of the reaction of olivine and pyroxene minerals with water (Onstott et al., 2019). Molecular hydrogen is also generated by water radiolysis, which also produces sulfate, an electron acceptor, via the oxidation of sulfides by radiolytically produced H_2_O_2_ (Lefticariu et al., 2006). A possible source of organic carbon for heterotrophic microorganisms in deep terrestrial biotopes is microbial necromass formed by chemolithotrophs. They can also use the mostly recalcitrant organic carbon associated with oil-, gas-, or coal-endowed sedimentary rocks (Kadnikov et al., 2020).

From an electron acceptor perspective, the deep environmental conditions below the Earth's surface have traditionally been considered anoxic and anaerobic (Lovley and Chapelle, 1995; Kieft et al., 2005; Liebensteiner et al., 2014). Sulfate, carbonate, iron, and manganese oxides were considered the most common electron acceptors (Onstott et al., 2019). However, a recent study demonstrated the presence of dissolved oxygen at concentrations of 0.52 ± 0.12 mg L^−1^ in multiple groundwater monitoring well samples (< 250 m depth) located in 14 aquifers in Canada (Ruff et al., 2023). It was assumed that the so-called “dark oxygen” formed in the absence of light arises from microbial dismutation of chlorite, nitric oxide, or H_2_O_2_, which is reflected in the isotopic composition of oxygen.

Metagenomic shotgun sequencing of water from a deep terrestrial aquifer in Mesozoic sediments at a depth of 2.0 km in Western Siberia revealed a plethora of organothrophic prokaryotes possessing aerobic respiratory pathways (Kadnikov et al., 2020). One of the MAG, designated as Ch19 and containing genes for cytochrome *c* oxidase, a marker of oxygen respiration, belonged to the class *Limnochordia*. The MAG made up 0.37% of the metagenome. In this study, we applied targeted isolation of the Ch19 bacterium using specific electron donors whose potential metabolism was inferred from the Ch19 MAG. Following the successful isolation of the Ch19 bacterium, another member of *Limnochordia* was isolated from a geographically distinct deep terrestrial aquifer in the Altay region based on the use of genomic information on the metabolism of *Limnochordia*. Here we report the isolation and characterization of two novel cultivated members of the class *Limnochordia* derived from different deep terrestrial thermal aquifers, capable of both aerobic and anaerobic respiration. One of the isolates exhibits a dual mode of life—heterotrophic by degradation of organic compounds and autotrophic by oxidation of CO and H_2_ using oxygen as an external electron acceptor.

## 2 Materials and methods

### 2.1 Deep water sampling and the initial enrichments set up

The geographical location, geology, and water characteristics of the deep boreholes used for cultivation have been previously described in detail (Kadnikov et al., 2017, 2018a, 2020; Lukina et al., 2023). Briefly, borehole 5P, which contains MAG Ch19 (Kadnikov et al., 2020), is a former oil-exploration artesian well located near the small village of Chazhemto in the Tomsk region of Western Siberia (58.0758 N, 82.8374 E). On July 11, 2019, water samples were collected from a sampling line at the wellhead for use in *Limnochordia* enrichments and subsequent pure culture isolation. Cells from 5 L of borehole water were collected on 0.22 μm cellulose nitrate membrane (Sartorius, Germany) using a Sartorius filtration unit.

Borehole 4E is located in the small town of Belokurikha in the Altay region (51.988832 N, 84.969378 E). The water from the borehole is used to supply mineral water to a local spa. On August 8, 2019, water samples for cultivation were collected from the wellhead of the 4E borehole. Both boreholes are classified as artesian wells; water flows from them under pressure, which minimizes the possibility of contamination from the surface. Water was not sampled until its physiochemical characteristics (temperature, pH, and Eh) were stable while flowing.

Water temperature, pH, and Eh were measured at the wellhead using a pH-meter HI 8314 (Hanna Instruments, Vöhringen, Germany) equipped with appropriate electrodes. The water sample was fixed with 2.4% Zn-acetate in a 1:5 proportion for hydrogen sulfide determination. H_2_S was measured colorimetrically in triplicate using a Smart Spec Plus spectrophotometer (Bio-Rad Laboratories, Hercules, CA) and the methylene blue method (Cline, 1969).

### 2.2 Pure culture isolation and characterization

Initial enrichments were prepared on-site. Cells from the water sample of the 5P borehole (Chazhemto) were concentrated by filtration through a 0.22 μm sterile filter (Sartorius, Germany). The total volume of filtered water was 5 L, and the filter was aseptically cut into 16 pieces, with each piece placed into a 500 ml serum bottle filled with cultivation medium. Initial enrichments were prepared in the modified spirochete basal medium (Karnachuk et al., 2021). The medium contained 2.0 g NaNO_3_, 1.0 g K_2_HPO_4_, 0.5 g MgSO_4_, 0.5 g KCl, 0.01 g FeSO_4_, 1.0 g yeast extract, and 1.0 g glucose per liter. It was also supplemented with 2 mL of vitamin solution (Widdel and Bak, 1992), 1 mL of selenite–tungsten solution (Widdel and Bak, 1992), and 1 mL of trace element solution (DSMZ141). Na_2_S·9H_2_O (96 g/L, 2 mL/L of basal medium) was used as a reducing agent, and each culture flask received an iron wire (100% Fe) as described previously (Karnachuk et al., 2019). Enrichments were incubated at 50°C. No preliminary cell concentration on the filter was used for the 4E (Belokurikha) borehole water sample. Instead, the 500-ml serum bottles with the modified spirochete medium were inoculated with approximately 50 ml of thermal water drawn directly from the borehole wellhead. Glucose was substituted with starch (1%). The pure culture was created using the serial dilution-to-extinction method. The purity of the cultures was determined through microscopic examination of bacterial morphology and genome sequencing. The 16S rRNA gene was amplified using the primer pairs 27F and 1492R (Lane, 1991) and sequenced commercially by Syntol Co. (Moscow, Russia) using the Sanger method.

Cell morphology was examined using both phase contrast microscopy (Axio Imager A1 microscope) and transmission electron microscopy (TEM) of ultra-thin sections, as described previously by Ikkert et al. (2013). Briefly, for TEM, cells from 500 ml serum bottles were harvested by centrifugation at 11,000 x g for 40 min and then washed with 1 x PBS buffer. Fixation of pelletized samples with glutaraldehyde, staining with osmium tetroxide, and dehydration with ethanol have been described previously by Ikkert et al. (2013). Ultra-thin sections (60–100 nm) were prepared using an ultramictome (Ultratome III, LKB, Stockholm, Sweden) and viewed with a JEM-100 CXII electron microscope (JEOL, Tokyo, Japan) at a voltage of 80 kV.

Growth experiments were conducted on a spirochete basal medium supplemented with vitamin, microelement, and selenite–tungsten solutions. Growth was tested at temperatures ranging from 28°C to 80°C and pH levels ranging from 5.0 to 10.0, and the pH was adjusted to the desired experimental pH values using 0.5 M H_2_SO_4_ or 2 M NaOH. The optimal salinity for growth was determined to be 0% to 2.5% NaCl. To test growth substrates for the LN^T^ strain, a modified DSMZ 963 medium was used, which contained (per liter): 0.78 g K_2_HPO_4_, 0.75 g KH_2_PO_4_, 0.04 g Na3 EDTA, 0.01 g FeSO_4_·7 H_2_O, 0.25 g MgSO_4_·7 H_2_O, 0.03 g CaCl_2_·2 H_2_O, 0.20 g NaCl, 0.050 g NH_4_Cl, 0.10 g yeast extract, 10 ml of Wolin's vitamin solution, 10 ml of DSMZ 318 trace element solution, and 5 ml of Na_2_S·9 H_2_O solution (Widdel and Bak, 1992). To test growth substrates for the L945^T^ strain, a modified Widdel-Bak (WB) medium (Widdel and Bak, 1992) was used, which contained (per liter): 0.15 g Na_2_SO_4_, 0.2 g KH_2_PO_4_, 0.25 g NH_4_Cl, 1.0 g NaCl, 0.4 g MgCl_2_·6H_2_O, 0.5 g KCl, 0.113 g CaCl_2_, 0.10 g yeast extract, 10 ml of Wolin's vitamin solution, 10 ml of DSMZ 318 trace element solution, and 2 ml of Na_2_S·9 H_2_O solution (Widdel and Bak, 1992). The strain growth was tested under anaerobic conditions with the following substrates: sucrose (2 mM), glucose (5 mM), fructose (5 mM), arabinose (10 mM), rhamnose (10 mM), maltose (10 mM), lactose (10 mM), mannose (10 mM), galactose (2 mM), sorbitol (10 mM), glycerol (10 mM), ethanol (20 mM), propanol (17 mM), formate (7.5 mM), pyruvate (7 mM), butyrate (7 mM), succinate (4.5 mM), lactate (7.3 mM), propionate (13.5 mM), acetate (10 mM), fumarate (10 mM), malate (7.5 mM), starch (1%), dextrin (1%), maltodextrin (1%), peptone (1%), gelatin (1%), chitin (2%), chitosan (2%), and microcrystalline cellulose (2%) (all Sigma-Aldrich). Fumarate (10 mM) was used as an electron acceptor. Insoluble substrates were added before autoclaving, and soluble substrates were then filtered through a 0.2 μm-pore-size filter and added into a sterilized medium. If growth was detected, the culture was sub-cultivated at least five times in the presence of each substrate to ensure proper utilization. For anaerobic cultivations, cultures were grown in liquid media in sealed culture vials with no headspace and incubated at the optimal temperature (65°C and 55°C for strains LN^T^ and L945^T^, respectively). Aerobic growth was assessed using pyruvate (7 mM) for strain LN^T^ and glucose (5 mM) for strain L945^T^. CO-fed cultures were grown in WB medium with N_2_ headspace using CO (1%−10%) as the sole electron donor. For aerobic cultivations, cultures were grown in 50-ml serum bottles with 70% gas phase under continuous agitation.

For the analysis of cellular fatty acids, biomass was harvested from cells grown in pyruvate (7 mM)- and fumarate-enriched (10 mM) spirochete basal medium until exponential growth phase, and 5 mg of freeze-dried cell material was treated with anhydrous 3N HCl/MeOH in PTFE-lined screw-cap borosilicate glass vial at 70°C for 2 h for conversion to fatty acid methyl ester. Cellular fatty acids (CFA) were determined by GC–MS (Thermo Scientific Trace GC Ultra DSQ II; CP-Sil 88 50 m × 250 μm × 0.20 μm highly polar cyanopropyl column; injection with a split ratio of 1:20; helium gas flow 1.2 mL/min; with a temperature program 5 min at 140°C, then 10 K/min to 220°C, then hold 22 min at 220°C; transfer line at 280°C EI 70 eV; mass scanning from 50 to 550 m/z at 0.25 scans/s) as a percentage of the total ion flow area.

### 2.3 Genome sequencing and analysis

Genomic DNA was isolated from strains LN^T^ and L945^T^ using a DNeasy PowerSoil DNA isolation kit (Mo Bio Laboratories, Carlsbad, CA, USA) and sequenced using Illumina and Oxford Nanopore technologies. For Illumina sequencing, the shotgun genome library was prepared using the NEBNext Ultra II DNA library prep kit (New England Biolabs, Ipswich, MA, USA) and sequenced on an Illumina MiSeq in a paired reads mode (2 × 300 nt). A total of 2,877,132 (1.3 Gb) and 3,786,751 (2.0 Gb) read pairs were generated for strains LN^T^ and L945^T^, respectively. Both genomes were additionally sequenced on a MinION instrument (Oxford Nanopore Technologies, Oxford, UK) using the ligation sequencing kit 1D and FLOMIN110 cells. A total of 663 Mb and 882 Mb of Nanopore sequences were obtained for strains LN^T^ and L945^T^, respectively.

The complete circular genome of strain LN^T^ was assembled from Nanopore reads with Flye v.2.8.2 (Kolmogorov et al., 2019). The consensus sequence of the assembled circular contig was corrected using Illumina reads and two iterations of Pilon v.1.22 (Walker et al., 2014). To obtain the complete circular genome of strain L945^T^, the hybrid assembly of Illumina and Nanopore reads was performed using SPAdes v.3.13.0 (Bankevich et al., 2012).

Gene search and annotation were carried out using the NCBI Prokaryotic Genome Annotation Pipeline (Tatusova et al., 2016) and the RAST server 2 (Brettin et al., 2015). AAI between the genomes was determined using the aai.rb script from the enveomics collection (Rodriguez-R and Konstantinidis, 2016). Prediction and comparative analysis of the metabolic capabilities of MAGs and genomes of isolates was performed using the Distilled and Refined Annotation of Metabolism (DRAM) tool (Shaffer et al., 2020). CheckM2 v.1.0.1 (Chklovski et al., 2023) was used to evaluate the completeness and contamination values of genomes. Genomes were taxonomically classified according to the genome-based taxonomic system (Parks et al., 2018) using the Genome Taxonomy Database Toolkit (GTDB-Tk) v.2.4.0 (Chaumeil et al., 2022) and Genome Taxonomy Database (GTDB), release 09-RS220.

For genome-based phylogenetic analysis, genes from all analyzed genomes were clustered using Blastclust v.2.2.26 of the BLAST package, with a minimum threshold of 40% alignment identity over 90% length of a shorter gene. One hundred and eighty single-copy genes presented in at least 24 of 29 genomes were selected based on the results of clustering. The alignment for the tree construction was created by concatenating alignments of each single-copy gene using MAFFT v.7 (Katoh and Standley, 2013). The concatenated alignment was additionally trimmed using ClipKit (Steenwyk et al., 2020) to remove errors and phylogenetically uninformative sites. A maximum-likelihood tree was computed by PhyML v. 3.3 (Guindon et al., 2010) using default parameters (LG amino acid substitution model, four substitution rate categories modeled by discrete gamma distribution with estimated shape parameter, and branch support values calculated using the approximate Bayes method). The default settings were used for all software unless specifically stated otherwise.

## 3 Results

### 3.1 5P and 4E boreholes water chemistry

The detailed water chemistry of the 5P and 4E boreholes has been reported in our previous studies (Kadnikov et al., 2017, 2018a; Lukina et al., 2023). Briefly, the 5P (Chazhemto) water temperature at the outflow varied from 14.5°C to 20.8°C, the water was anoxic (Eh from −329 to −480 mV), and the pH was circumneutral (7.5–7.6). At the time of sampling for cultivation on July 11, 2019, the temperature was 14.5°, the pH was 7.5, and the Eh was −420 mV. The H_2_S concentration was 3.46 ± 0.47 mg/l. The 4E (Belokurikha) thermal water was slightly anoxic and alkaline. At the time of sampling for cultivation on 8 August 2019 the temperature was 38.0°C, pH −9.12 and Eh −40 mV.

### 3.2 MAG-based isolation of uncultivated *Limnochordia*

Microscopic observation of the set of enrichments from the 5P (Chazhemto) borehole revealed different morphotypes, including vibrios, rods, filamentous cells, and long rods with pointed ends (Supplementary Figure S1). Based on the previous reports on the cell morphology of *L. pilosa* (Watanabe et al., 2015, 2016), it was suggested that the long rods may represent the Ch19 *Limnochordia* bacterium, which MAG has been previously assembled from the 5P (Chazhemto) borehole water (Kadnikov et al., 2020). This MAG provided a clue for the enrichment and selection of the Ch19 bacterium. The sorbitol degradation pathway was present in the genome, making sorbitol the chosen growth substrate for the *Limnochordia* bacterium pure culture isolation. The pure culture was obtained using the serial dilution-to-extinction method on the modified spirochete medium with sorbitol (10 mM). The strain was designated LN^T^, and its 16S rRNA gene sequence analysis showed that it represented a distinct lineage with a sequence similarity with that of only 85.2% to that of *L. pilosa* HC45^T^.

Considering the amylolytic potential predicted by analysis of the Ch19 bacterium MAG (Kadnikov et al., 2020), starch (1%) was chosen as the growth substrate for the isolation of the *Limnochordia* bacterium from another deep thermal borehole, 4E in Belokurikha. *Limnochorda*-like morphotypes were observed in the enrichment culture with starch, and the bacteria formed spherical bodies. Given the small width of the *Limnochordia*-like morphotypes, filtration through a 0.22 μm filter was chosen for further enrichment. After a series of dilutions, a pure culture isolate was obtained and designated strain L945^T^. The 16S rRNA gene sequence showed that *L. pilosa* was the closest cultivated and validly described species with a sequence similarity of 87.05%.

### 3.3 Morphology, growth conditions and physiology of *Geochorda subterranea* gen. nov. sp. nov. LN^T^ and *Carboxydochorda subterranea* gen. nov. sp. nov. L945^T^

**Figure 1:**
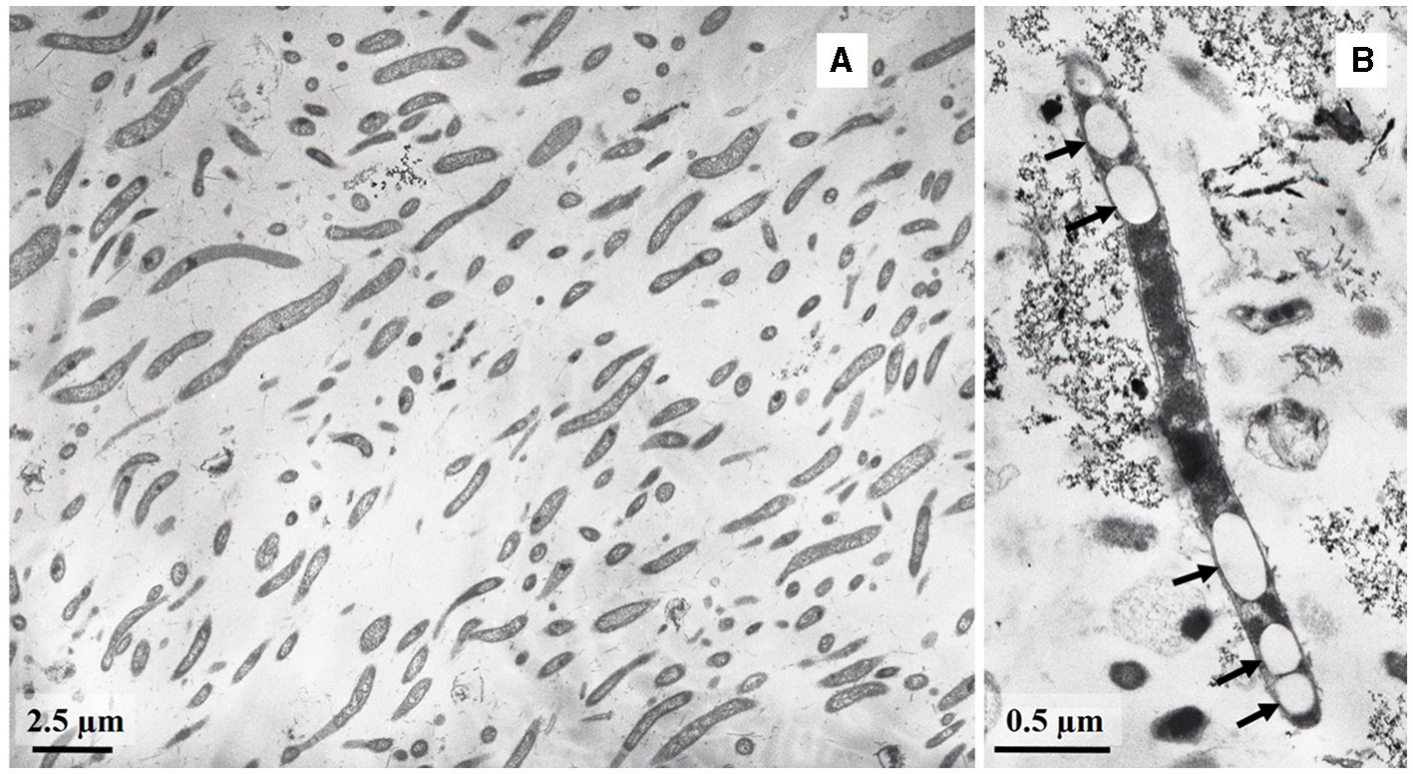
TEM micrographs of ultra-thin sections of strain LN^T^ cells **(A)** and cells containing electron-transparent granules (shown by arrows) **(B)**.

**Figure 2:**
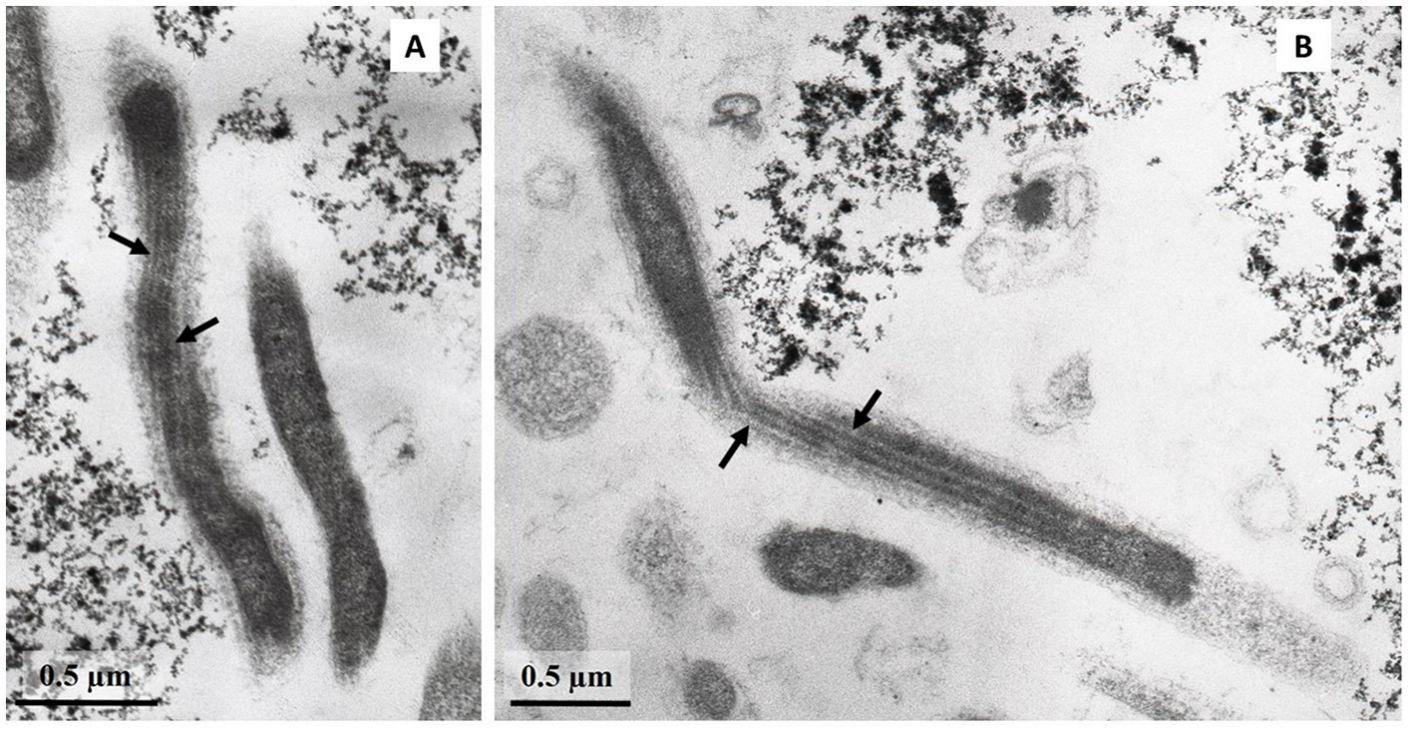
**(A, B)** TEM micrographs of ultra-thin sections of strain LN^T^ with filamentous electron-transparent structures (shown by arrows).

**Figure 3:**
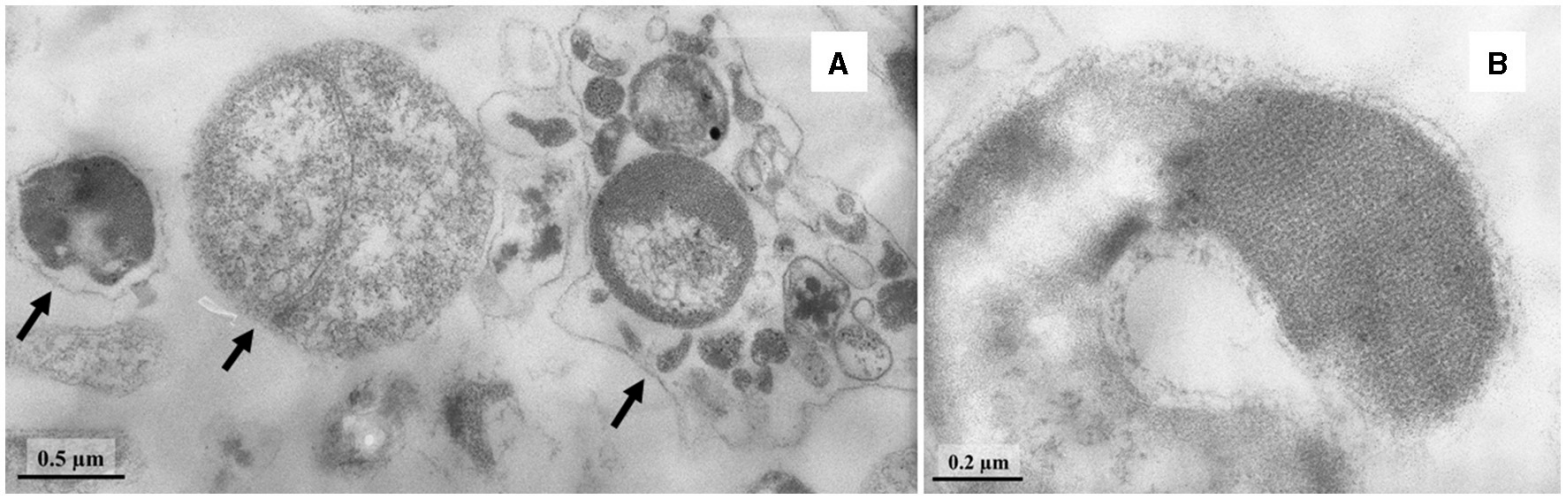
TEM micrographs of ultra-thin sections of round bodies (shown by arrows) **(A)** and S-layer **(B)** of strain LN^T^.

Cells of strain LN^T^ were curved rods, 1.5–9.0 μm long and 0.15–0.20 μm wide (Figure 1). The cells were immotile under all studied culture conditions, despite the presence of a set of genes necessary for flagellar motility and chemotaxis in the genome. TEM micrographs of ultra-thin sections of cells revealed filamentous electron-transparent structures (Figure 2). The cells had a didermic (Gram-negative) wall containing an outer membrane (Figure 2) and formed spherical bodies (Figure 3A). In cells and spherical bodies grown with pyruvate (9 mM) and fumarate (1 mM) for 9 days, an S-layer was observed (Figure 3B). The genome of the strain LN^T^ contains an S-layer homology domain-containing protein, which is an amino acid sequence that shares only 27.4% identity with that of *L. pilosa* but 50.1% with uncultivated *Limnochordales* MAGs binned from burning coal seams (Bu05) (Kadnikov et al., 2023). Electron-lucent granules were also observed in cells grown with pyruvate and fumarate for 7 days (Figure 1).

**Figure 4:**
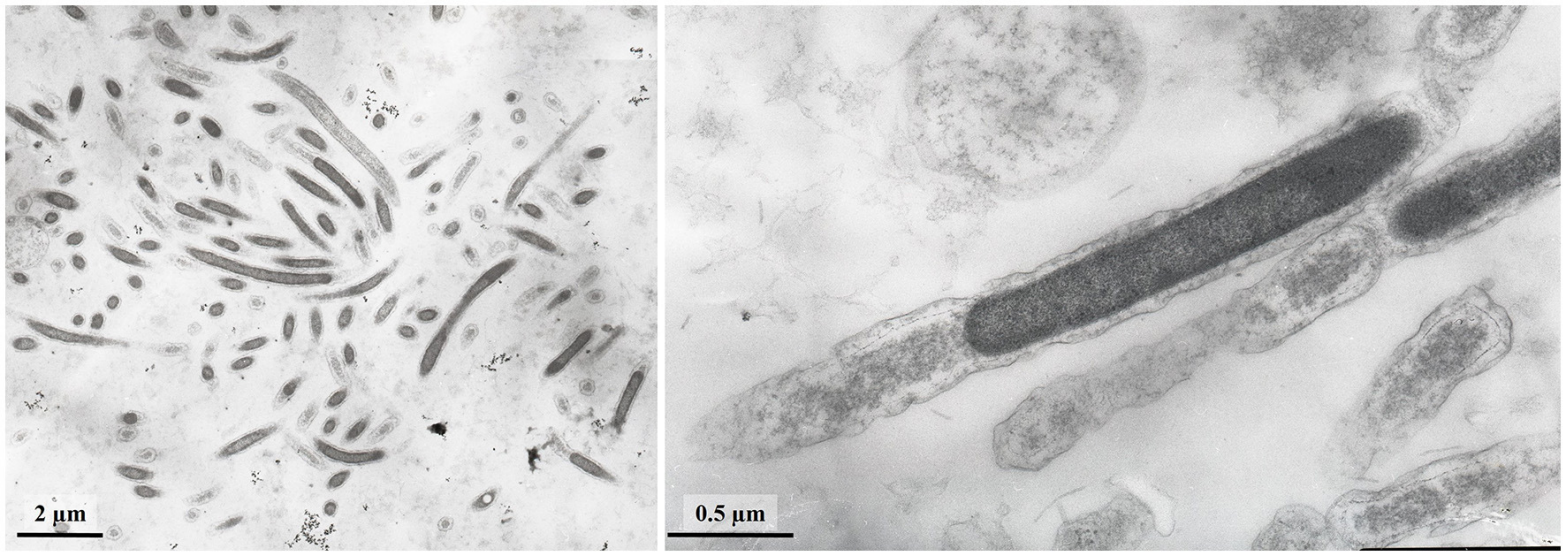
TEM micrographs of ultra-thin sections of strain L945^T^ cells.

**Figure 5:**
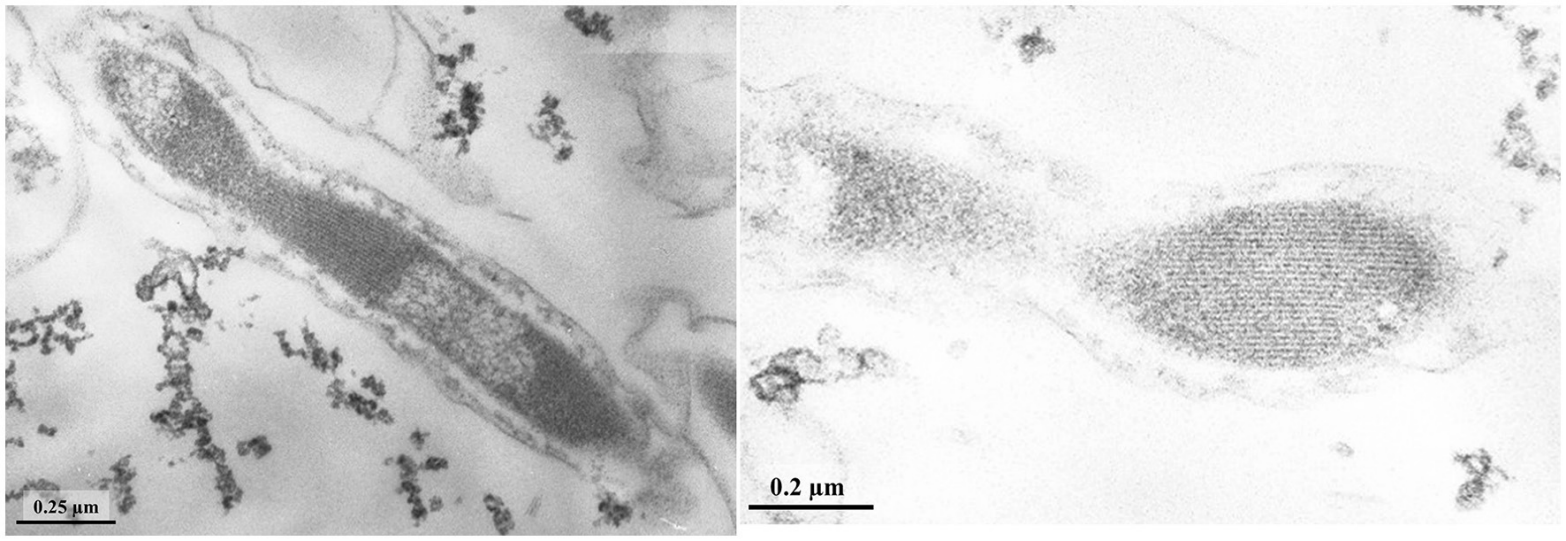
TEM micrographs of ultra-thin sections of strain L945^T^ cells with S-layer.

Strain L945^T^ cells reached lengths of 1.5 to 7.0 μm and widths of 0.15–0.30 μm (Figure 4). The strain formed round bodies of up to 2 μm in size. The cell wall of the strain was didermic (Gram-negative), containing an outer membrane and S-layer, which was visible in old cultures grown for 50 days (Figure 5). However, no S-layer proteins were found in the genome.

Strain LN^T^ had a relatively wide pH growth range of pH 6.0 to 9.5, with an optimum of 7.5–8.0 and necessary NaCl for growth. The optimal NaCl range for growth varied from 0.1% to 2.0% (w/v), with an optimum of 1.0%−1.5% (w/v). The temperature range for the strain growth was 45°−70° with an optimum of 65°. The highest growth rate was observed when strain LN^T^ was grown with pyruvate and fumarate. Fumarate respiration was also observed with a narrow range of sugars, including fructose, glucose, sucrose, galactose, and propionate. The strain was able to hydrolyze starch, dextrin, maltodextrin, xylan, chitin, and chitosan in the presence of fumarate in the medium. Slow growth was observed when sorbitol and malate were used as electron donors for fumarate respiration. The strain was unable to use arabinose, rhamnose, maltose, lactose, mannose, glycerol, ethanol, propanol, formate, butyrate, succinate, lactate, acetate, peptone, gelatin, tryptone, soytone, or microcrystalline cellulose for fumarate respiration. Strain LN^T^ did not use any of the tested inorganic electron acceptors for anaerobic respiration, including sulfate, sulfite, thiosulfate, elemental sulfur, nitrate, nitrite, or Fe–NTA. Surprisingly, strain LN^T^, isolated from the deep subsurface, was able to grow aerobically respiring with O_2_ using glucose, fructose, or sucrose as electron donors. However, the strain could not grow on glucose, lactate, or pyruvate without adding fumarate to the medium. No autotrophic growth was observed with formate or CO as the electron donor and fumarate or O_2_ as the electron acceptor. Also, the strain did not grow with H_2_ and CO_2_.

The fatty acid profile of the LN^T^ strain cell wall indicated that the major proportion (>10%) comprised iso-C15:0 (23.1%), anteiso-C17:0 (18.4%), iso-C16:0 (16.3%), anteiso-C15:0 (15.0%), and C16:0 (13.6%) (Supplementary Table S1).

Strain L945^T^ could grow at pH 6.5–9.0, with an optimum of 7.0–7.5. The optimal growth was observed without NaCl addition to the growth medium. The strain could tolerate up to 1% NaCl. The temperature range for strain L945^T^ was 37°−60° with an optimum of 55°. Similar to strain LN^T^, strain L945^T^ utilized pyruvate, fructose, glucose, sucrose, and galactose with fumarate as an electron acceptor. The strain was unable to hydrolyze dextrin and maltodextrin in the presence of fumarate in the medium. Slow growth with chitin occurred in the presence of fumarate in the medium. The strain did not use arabinose, rhamnose, maltose, lactose, mannose, glycerol, ethanol, propanol, butyrate, succinate, lactate, propionate, acetate, peptone, gelatin, or microcrystalline cellulose for fumarate respiration. Similar to strain LN^T^, L945^T^ grew under aerobic conditions using glucose, fructose, and sucrose as electron donors for aerobic respiration with O_2_. In addition to organic electron donors, L945^T^ was capable of autotrophic growth with CO (2%) or formate with O_2_ as an electron acceptor. CO was also used as an electron donor for fumarate respiration. Sulfate, sulfite, thiosulfate, nitrate, nitrite, or Fe–NTA were not used by the strain as electron acceptors. However, strain L945^T^ actively grew by sulfur respiration with glucose as an electron donor.

Lipid analysis indicated that similar to *L. pilosa* (Watanabe et al., 2015), the major fatty acids (>10%) present in strain L945^T^ cell wall were anteiso-C15:0 (46.1%) and iso-C15:0 (28.3%) (Supplementary Table S1).

### 3.4 Genome-based phylogenetic placement of strains LN^T^ and L945^T^

**Figure 6:**
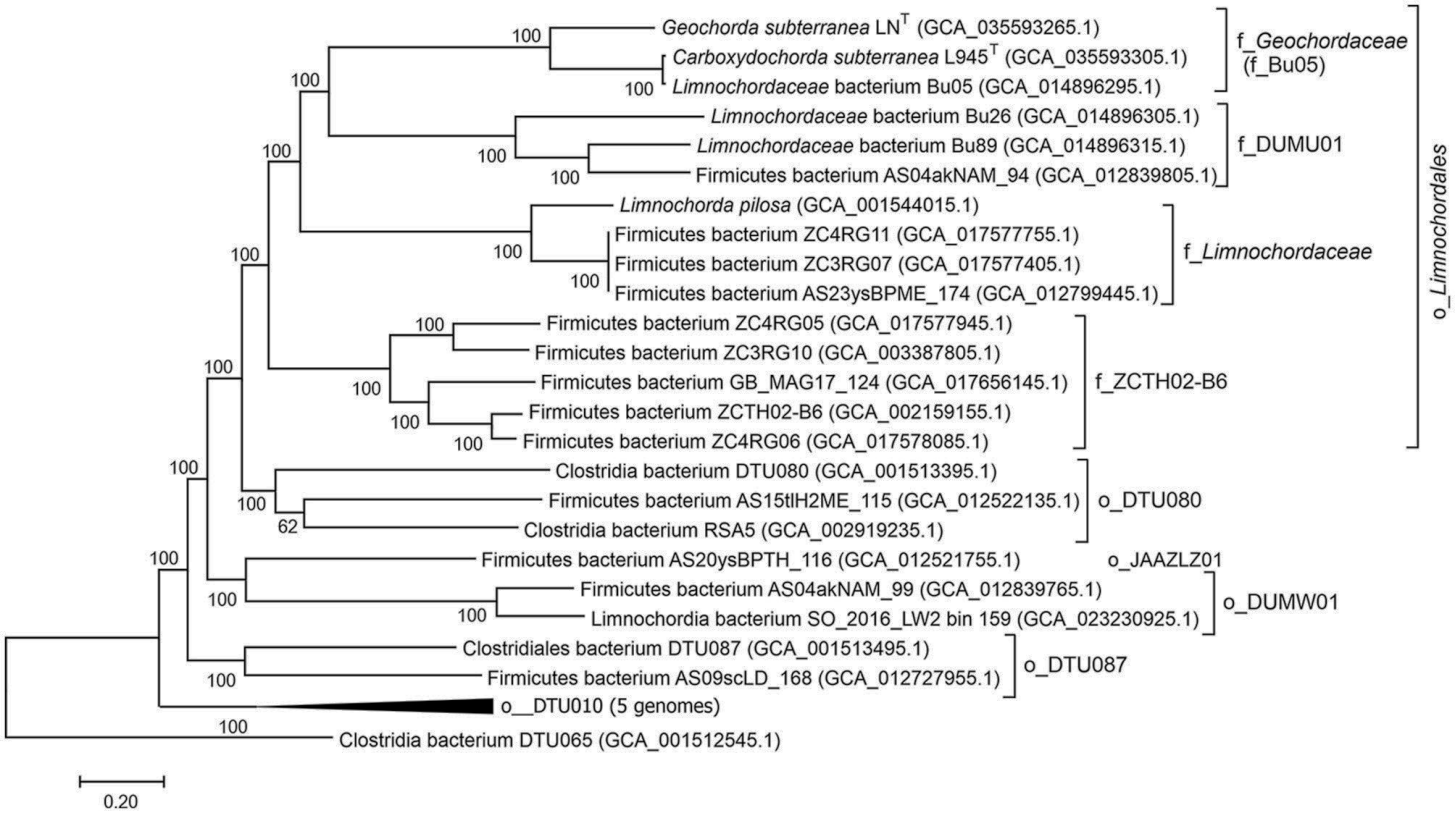
Genome-based phylogeny of *Limnohordia*. The level of support for internal branches was assessed using the Bayesian test in PhyML. Taxonomy is shown according to the GTDB release R207 (o, order; f, family). The genome of Clostridia bacterium DTU065 representing a sister class DTU065 in the candidate phylum Firmicutes G was used to root the tree.

Taxonomic assignment of strains LN^T^ and L945^T^ was determined by searching against the Genome Taxonomy database (GTDB) (Parks et al., 2018), placing them in the phylum *Firmicutes (Bacillota)*, class *Limnochordia*, order *Limnochordales*, and family Bu05. To further characterize the phylogeny of the class *Limnochordia*, we constructed a phylogenetic tree based on the concatenated sequences of 180 conserved marker genes, including the LN^T^ and L945^T^ genomes, 13 other genomes from the order *Limnochordales*, and 13 genomes representing other candidate orders of *Limnochordia* (Figure 6). All lineages recognized by the GTDB within the *Limnochordia* were represented by distinct monophyletic branches.

The average amino acid sequence identity between the LN^T^ and L945^T^ genomes was 67.13%, which is above the proposed genera delineation threshold (65%, Konstantinidis et al., 2017). However, their 16S rRNA gene sequences were 93.51% identical, suggesting that they represent different genera. The closest relative of L945^T^, with an AAI of 97.13%, is an uncultivated *Limnochordaceae* bacterium Bu05 (GCA_014896295.1), obtained from the coal-fire heated soil in Eastern Siberia (Kadnikov et al., 2023). Apparently, these bacteria belong to the same species.

### 3.5 Main metabolic pathways

**Figure 7:**
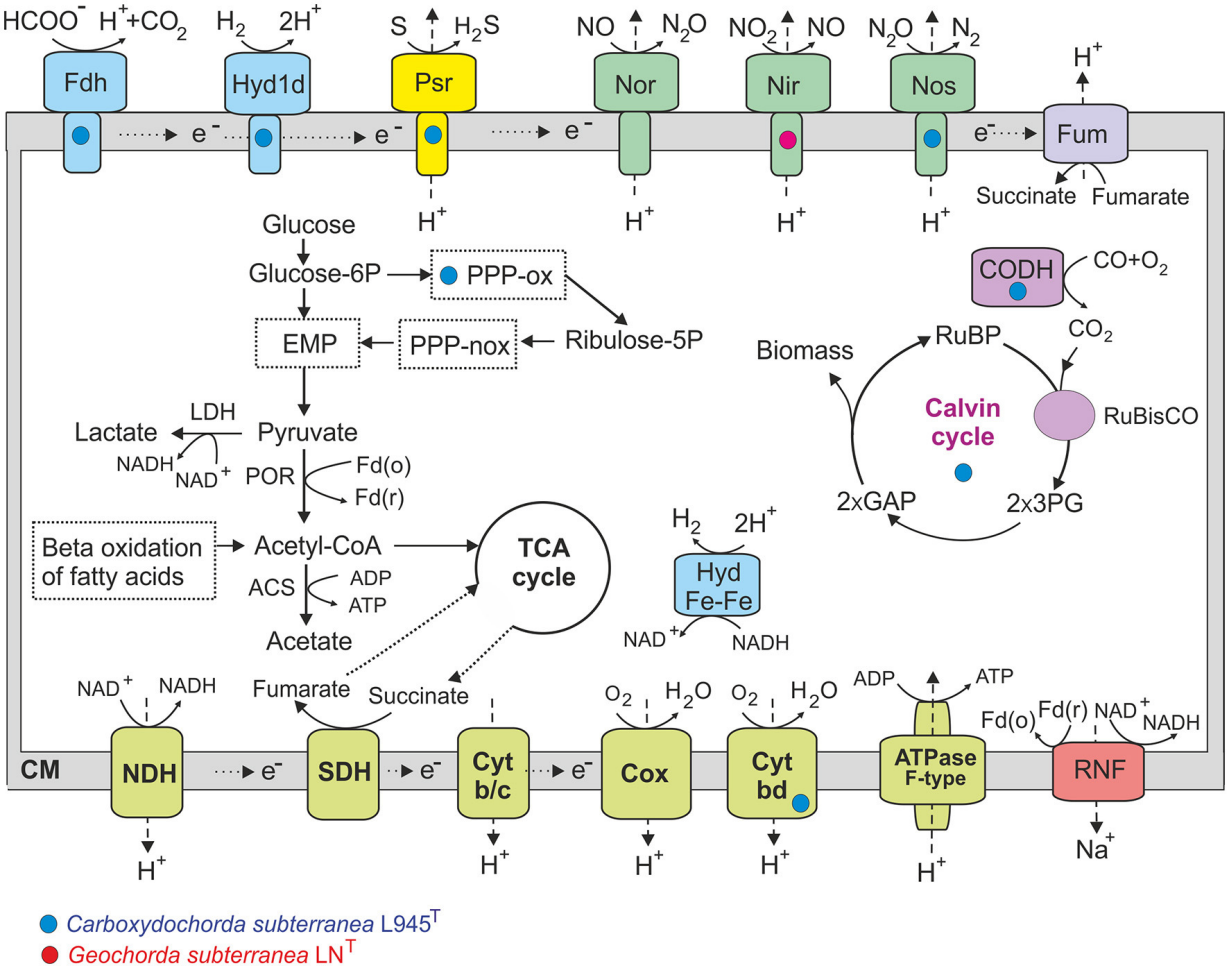
An overview of metabolic pathways of *Geochorda subterranea* LN^T^ and *Carboxydochorda subterranea* 945^T^. Genes/pathways present only in one strain are marked by red (in LN^T^) or blue (in 945^T^) circles, other traits were found in both genomes. EMP, Embden–Meyerhof pathway; PPP-ox and PPP-nox, oxidative and non-oxidative phases of the pentose phosphate pathway; POR, pyruvate ferredoxin oxidoreductase; ACS, acetyl-CoA synthetase; LDH, lactate dehydrogenase; NDH, NADH dehydrogenase; SDH, succinate dehydrogenase; Cyt b/c, cytochrome *b/c* complex; Cox, cytochrom *c* oxidase; Cyt bd, quinol oxidase *bd* complex; Fdh, formate dehydrogenase; Hyd1d, membrane-bound [NiFe] group 1d uptake hydrogenase; Hyd Fe-Fe, cytoplasmic [Fe-Fe] group A hydrogenase; Psr, polysulfide/tetrathionate reductase; Nor, nitric-oxide reductase; Nir, nitrite reductase; Nos, nitrous-oxide reductase; Fum, fumarate reductase; CODH, carbon monoxide dehydrogenase; Glucose-6P, glucose 6-phosphate; Ribulose-5P, ribulose 5-phosphate; RuBP, ribulose-1,5-bisphosphate; 3PG, 3-phosphoglycerate; GAP, glyceraldehydes-3-phosphate; Fd(o)/Fd(r), ferredoxin, oxidized and reduced form.

Analysis of the LN^T^ and L945^T^ genomes revealed the presence of a complete set of genes encoding enzymes for the Embden–Meyerhof glycolytic pathway, gluconeogenesis, and the tricarboxylic acid (TCA) cycle (Figure 7). Both genomes also contain genes encoding the malic enzyme, which decarboxylates malate to pyruvate, and the phosphoenolpyruvate carboxylase, which catalyzes the addition of bicarbonate to phosphoenolpyruvate to form oxaloacetate. The strain L945^T^ genome encodes the pentose phosphate pathway, including both oxidative and non-oxidative branches, while only the latter is encoded in the LN^T^ strain. Pyruvate produced in the glycolysis could be decarboxylated to acetyl coenzyme A (CoA) by pyruvate:ferredoxin oxidoreductase or converted to lactate by lactate dehydrogenase. The formation of acetate as a fermentation product can be enabled by acetyl-CoA synthetase (ADP-forming).

All major components of the aerobic respiratory chain are encoded in both genomes, including the proton-translocating NADH:quinone oxidoreductase, membrane-linked succinate dehydrogenase, cytochrome *bc* complex III, and terminal oxidases. Both genomes also encode two proton-translocating cytochrome *c* oxidases, and the L945^T^ genome additionally harbors genes for cytochrome *bd* ubiquinol oxidase. Physiological experiments confirmed the ability of both strains to respire O_2_. The ability of both bacteria to grow aerobically is consistent with the presence of superoxide dismutase and catalase participating in protection against oxidative stress in aerobes.

Genome analysis revealed no known reductases for dissimilatory reduction of sulfate and other sulfur compounds, nitrate, and arsenate, in consistent with the observed lack of growth with ${\text{SO}}_{4}^{2-}$ and ${\text{NO}}_{3}^{-}$. In addition to fumarate reductase, which enables anaerobic respiration with fumarate, the other terminal reductases for anaerobic respiration are the nitric-oxide reductase, found in genomes, copper-containing nitrite reductase in strain LN^T^, and nitrous-oxide reductase in strain L945^T^. However, we were unable to grow LN^T^ and L945^T^ with nitrite. Additionally, the strain L945^T^ genome was found to contain genes for the complex iron–sulfur molybdoenzyme oxidoreductase of the Psr/Phs family, which could perform the reduction of sulfur and/or tetrathionate. Consistently, strain L945^T^ showed active growth with elemental sulfur.

The transmembrane proton gradient generated by respiratory chains may be used for ATP synthesis by an F_0_F_1_-type ATPase. Both bacteria have an additional mechanism to generate a transmembrane ion gradient that can be used for ATP synthesis, namely the ion-transporting complex Rnf (*rnfCDEAB* genes). In *Firmicutes*, this membrane-linked complex acts catabolically as a ferredoxin: NAD+ oxidoreductase, moving sodium ions or protons across the membrane out of the cell (Biegel et al., 2011).

One important difference between strains LN^T^ and L945^T^ is the presence of key genes involved in the Calvin–Benson–Bassham cycle for autotrophic carbon fixation in the latter, including ribulose bisphosphate carboxylase and phosphoribulokinase. The Wood–Ljungdahl pathway was not found in both genomes, as well as reductive TCA, as evidenced by the absence of citrate lyase. Thus, CO_2_ fixation in L945^T^ growth experiments with H_2_ and CO_2_ presumably occurs via the Calvin–Benson–Bassham cycle.

The genome of strain L945^T^ also contains the genes for membrane-bound formate dehydrogenase and a membrane-linked [NiFe] group 1d hydrogenase, which enable hydrogenotrophic respiration using O_2_ or fumarate as terminal electron acceptors (Greening et al., 2016). These oxygen-tolerant hydrogenases are commonly found in *Firmicutes*. Aerobic carbon monoxide dehydrogenase genes were also found in the genome of this strain, which is consistent with its observed ability to grow autotrophically on CO in the presence of oxygen. These traits were absent in strain LN^T^.

In the search for potentially secreted glycosyl hydrolases that could enable the utilization of polysaccharides, a limited set of enzymes containing N-terminal secretion signal peptides was found. Both genomes contain genes for the utilization of chitin and N-acetyl-D-glucosamine (GlcNAc), including GH18 family chitinases and GH3 family beta-N-acetylglucosaminidases. These enzymes carry N-terminal secretion signals, suggesting their extracellular operation in the hydrolysis of chitin and chitooligosaccharides. The coordinated activity of these enzymes could result in the hydrolysis of chitin into oligomers and the production of GlcNAc monomers, which may be imported by ATP-binding cassette (ABC)-type transporters. In the cytoplasm, GlcNAc may be phosphorylated by *N*-acetylglucosamine kinase. Then, GlcNAc-6-P could be deacetylated by *N*-acetylglucosamine-6-phosphate deacetylase to form glucosamine-6-phosphate. The latter is then converted by glucosamine-6-phosphate deaminase into fructose-6-P, which enters the Embden–Meyerhof pathway (Beier and Bertilsson, 2013).

Consistent with the observed ability of strain LN^T^ to hydrolyze starch, its genome comprises an operon including genes for secreted GH57 family alpha-amylase, maltodextrin ABC transporter, and maltodextrin glucosidase of the GH13 family. Strain L945^T^ also harbors a similar operon, but it lacks GH57 alpha-amylase. Both genomes also encode several other GH13 and GH57 hydrolases that lack N-terminal signal peptides.

The LN^T^ genome encodes an endo-1,4-beta-xylanase of the GH10 family and an enzyme of the GH16 family with different activities, including endo-xyloglucanase and endo-β-1,3-glucanase/laminarinase. This genome also contains genes for ABC transporters of beta-xylosides and xylose, as well as intracellular enzymes involved in xylan metabolism, such as GH39 family beta-xylosidase, xylose isomerase, and xylulose kinase. Growth of the strain with xylan was observed under anaerobic conditions in the presence of fumarate in physiological experiments. However, strain L945^T^ lacked extracellular xylanolytic enzymes. On the contrary, both genomes encode numerous ABC-type transporters for the import of simple sugars, including galactose, maltose, maltodextrin, and alpha-glucosides, as well as transporters for amino acids, oligo-, and dipeptides. The presence of an L-rhamnose ABC transporter, L-rhamnose isomerase, and rhamnulokinase in the strain L945^T^ genome suggests that it can utilize rhamnose. However, this was not confirmed in physiological experiments.

In addition to transporters for sugars and amino acids, both genomes contain several transport systems that enable the uptake of di- and tricarboxylates of the TCA cycle. These include the tripartite tricarboxylate transporters TctABC for citrate (Rosa et al., 2018) and the TRAP-type C4-dicarboxylate transport system, enabling the symport of C4-dicarboxylates (succinate, fumarate, and malate) and aspartate with sodium ions or protons (Janausch et al., 2002). Both strains appear to be able to use fatty acids as growth substrates, as evidenced by the presence of a fatty acid beta-oxidation pathway. This includes genes for long-chain fatty acid-CoA ligases, acyl-CoA dehydrogenases, enoyl-CoA hydratases, 3-hydroxyacyl-CoA dehydrogenases, and 3-ketoacyl-CoA thiolases.

### 3.6 Description of *Geochorda* gen. nov.

*Geochorda* (Ge.o.chor′da. Gr. fem. n. gê the Earth; L. fem. n. *chorda*, chord, string; N.L. fem. n. *Geochorda* Earth string).

Cells are curved rods 0.15–0.20 μm in diameter and up to 9.0 μm long. These cells stain Gram-negative, have a didermic cell wall, and form round bodies up to 1.5 μm in diameter. They are facultatively anaerobic, thermophilic, and chemoorganoheterotrophic and are capable of hydrolyzing polysaccharides, including starch, dextrin, maltodextrin, and xylan. They are also free-living.

The type species is *Geochorda subterranea*.

### 3.7 Description of *Geochorda subterranea* sp. nov.

*Geochorda subterranea* (sub.ter.ra′ne.a. L. prep. *sub*, below underneath; L. fem. n. *terra*, soil; L. fem. adj. *subterranea* below the terrestrial ground, referring to the place of isolation).

Exhibit the following properties in addition to those listed in the genus description. The temperature range for growth is 45°C−70°C, with an optimum of 65°C. The pH range for growth is 6.0 to 9.5, with an optimum of 7.5–8.0. NaCl is necessary for growth, with an optimal concentration of 1.0%−1.5% (w/v). Fructose, glucose, sucrose, galactose, and propionate serve as electron donors during fumarate respiration. Hydrolyzes starch, dextrin, maltodextrin, xylan, chitin, and chitosan in the presence of fumarate in the medium. Glucose, fructose, or sucrose are used as electron donors for aerobic respiration. Arabinose, rhamnose, maltose, lactose, mannose, glycerol, ethanol, propanol, formate, butyrate, succinate, lactate, acetate, peptone, gelatin, or microcrystalline cellulose are not used as electron donors. Sorbitol and malate support slight growth, with fumarate as an electron acceptor. Sulfate, sulfite, thiosulfate, elemental sulfur, nitrate, nitrite, or Fe–NTA do not serve as electron acceptors. The major fatty acids are iso-C_15:0_, anteiso-C_17:0_, iso-C_16:0_, anteiso-C_15:0_, and C_16:0_. The species description is based on the properties of strain LN^T^ (=VKM B-3703^T^ = JCM 39508) isolated from a deep subsurface aquifer in Chazhemto (Tomsk region, Russia). The DNA G+C content is 70.96 mol% (genome).

### 3.8 Description of *Carboxydochorda* gen. nov.

*Carboxydochorda* (Car.bo.xy.do.chor′da. N.L. neut. N. *carboxydum* carbon monoxide; L. fem. n. *chorda* chord, string; N.L. fem. n. *Carboxydochorda* carbon monoxide using string).

Cells are curved rods measuring 0.15–0.30 μm in diameter and up to 7.0 μm in length. Gram-negative cells have a didermic cell wall and round bodies measuring 0.7–1.5 μm in diameter. These are facultatively anaerobic, moderately thermophilic, and chemoorganoheterotrophic or chemolithoautotrophic. CO acts as an electron donor during fumarate or aerobic respiration. They are also free-living.

The type species is *Carboxydochorda subterranea*.

### 3.9 Description of *Carboxydochorda subterranea* sp. nov.

*Carboxydochorda subterranea* (sub.ter.ra′ne.a. L. prep. *sub*, below, underneath; L. fem. n. *terra*, soil; L. fem. adj. subterranea below the terrestrial ground, referring to the place of isolation).

Exhibit the following features in addition to those specified in the genus description. The temperature range for growth is 37°C−60°C, with an optimum of 55°C. The pH range for growth is 6.5–9.0, with an optimum of 7.0–7.5. This does not require NaCl for growth. Fructose, glucose, sucrose, and galactose are used as electron donors for fumarate respiration. Glucose, fructose, sucrose, H_2_, CO, and formate are used as electron donors for aerobic respiration. Arabinose, rhamnose, maltose, lactose, mannose, glycerol, ethanol, propanol, formate, butyrate, succinate, lactate, acetate, peptone, gelatin, dextrin, maltodextrin, and microcrystalline cellulose are not used as electron donors. This hydrolyzes chitin and chitosan in the presence of fumarate. Sulfate, sulfite, thiosulfate, nitrate, nitrite, or Fe–NTA do not serve as electron acceptors. Elemental sulfur is used as an electron acceptor in the presence of glucose in the medium. The main fatty acids are anteiso-C_15:0_ and iso-C_15:0_. The species description is based on the properties of strain L945^T^ (= VKM B-3704^T^ = JCM 39509), which was isolated from a deep subsurface aquifer in Belokurikha (Altay region, Russia). The DNA G+C content is 69.48 mol% (genome).

### 3.10 Description of *Geochordaceae* fam. nov.

*Geochordaceae* (Ge.o.chor'da.ce'ae. N.L. fem. n. *Geochorda*, a bacterial genus; –*aceae*, ending to denote a family; N.L. fem. pl. n. *Geochordaceae*, the family of *Geochorda* genus).

Cells are curved rods, stain Gram-negative, have didermic cell walls, and form round bodies. These are facultatively anaerobic, thermophilic, and chemoorganoheterotrophic or chemolithoautotrophic. They are also capable of hydrolyzing polysaccharides, including starch, dextrin, maltodextrin, xylan, and chitin. They are also free-living.

The family belongs to the *Limnochordales* order.

The type genus is *Geochorda* gen. nov.

### 3.11 Comparative genomics of *Limnochordales*

At present, in addition to *L. pilosa*, strains LN^T^ and L945^T^, the order *Limnochordales* comprises nine uncultivated species defined on the basis of the corresponding genomes (Figure 6). In order to gain insights into the lifestyle of *Limnochordales*, we conducted a comparative genomic analysis of its representatives and analyzed the presence of important metabolic pathways using the DRAM tool. Since only three genomes derived from cultivated isolates (*L. pilosa*, strains LN^T^ and L945^T^) were assembled as closed circular chromosomes and are assumed to be complete the absence of certain genes/pathways in the remaining MAGs should be considered with caution, as it may be due to their incompleteness. The completeness and contamination of the genomes, according to the CheckM2 estimates, are shown in Supplementary Table S2.

**Figure 8:**
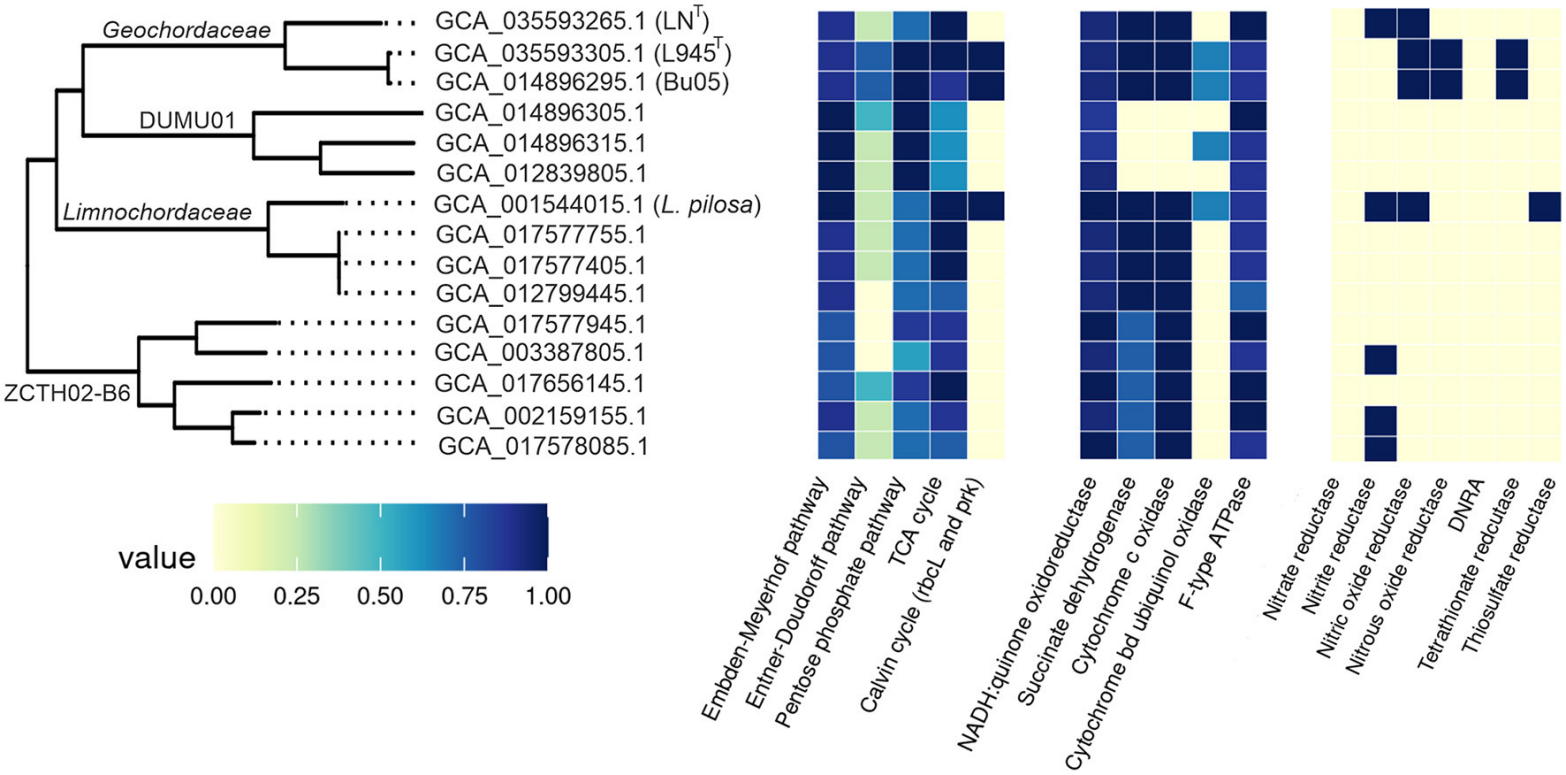
Comparative analysis of the main metabolic pathways along the *Limnochordales* (shaded in blue) and Limnochordales_A (shaded in green) using the DRAM tool. Strain/MAG names discussed in the text are shown after genome accession numbers. DNRA, ammonia-forming cytochrome c nitrite reductase.

All members of *Limnochordales* possess most of the genes of the Embden–Meyerhof pathway and the non-oxidative stage of the pentose phosphate pathway, while the Entner–Doudoroff pathway is absent (Figure 8). Cytochrome c oxidases were found in all genomes except for three MAGs representing the family DUMU01. All these enzymes are low-affinity *aa3*-type oxidases; the high-affinity *cbb3*-type enzymes were not found. Additionally, DUMU01 genomes also lacked genes enabling anaerobic respiration and the complete TCA cycle, indicating that DUMU01 bacteria are devoted to a fermentative lifestyle.

Some members of *Limnochordales* possess genes enabling anaerobic respiration with nitrogen compounds, such as NO-forming nitrite reductase (*nirK*), nitrous-oxide reductase, and nitric-oxide reductase, which were found in five, two, and four genomes, respectively. However, genes for dissimilatory nitrate reductase and ammonia-forming cytochrome c nitrite reductase (*nrfAH*) were not found in any of the genomes. The ability to perform dissimilatory reduction of oxidized sulfur compounds appears to be rare. None of the genomes encodes the dissimilatory sulfate reduction pathway. Tetrathionate and thiosulfate reductases were identified in two (including strain L945^T^) and one genome, respectively.

The ribulose bisphosphate carboxylase and phosphoribulokinase genes, which are hallmarks of the Calvin cycle for autotrophic carbon fixation, were found in the genomes of *L. pilosa*, strain L945^T^ and the MAG Bu05. All three genomes also contain genes for formate dehydrogenase and [NiFe] group 1d hydrogenase, while aerobic carbon monoxide dehydrogenase genes were only found in strains L945 ^T^ and *L. pilosa*. These genes are absent in other *Limnochordales* genomes. Probably only these three members of *Limnochordales* are capable of chemolithoautotrophic growth. Furthermore, the ribulose bisphosphate carboxylase and phosphoribulokinase from these three bacteria are more similar to each other in amino acid sequences than to proteins from other microorganisms, indicating that the Calvin cycle genes were acquired by the common ancestor of *Limnochordales* through a single horizontal transfer event and were subsequently lost in most lineages. The absence of the Calvin cycle in strain LN^T^, the closest relative of the strain L945^T^/Bu05 group, supports this hypothesis well.

Thus, the ability for heterotrophy and aerobic respiration is likely the most common and ancestral trait in the *Limnochordales*. Respiratory capacities were lost only in family DUMU01. The ability for autotrophic CO_2_ fixation and the use of hydrogen and CO as energy sources were apparently acquired later and then lost in most phylogenetic lineages.

## 4 Discussion

Until now, the class *Limnochordia* was represented by a single cultivated member, *L. pilosa*, isolated from meromictic lake sediments (Watanabe et al., 2015), and our knowledge of this phylogenetic group was based on the uncultivated majority, primarily from sludge samples (Taib et al., 2020). Using high-throughput sequencing, we discovered the presence of a member of this class in water samples from the deep subsurface thermal aquifer, broached by borehole 5-P (Chazhemto), where the *Limnochordia* phylotype (MAG Ch19) was not abundant and constituted < 1% of the prokaryotic community (Kadnikov et al., 2020). The metabolic features of *Limnochordia* inferred from genomic data were also used to enrich and isolate a cultivated representative of the class from a geographically distant deep thermal aquifer, broached by borehole 4E in Belokurikha (Altay Region). The latter deep thermal water was previously distinguished by the simultaneous presence of both cultivated anaerobic and aerobic prokaryotes (Lukina et al., 2023). The boreholes sampled in this study, 5-P in Chazhemto and 4E in Belokurikha, are artesian wells with pressurized water outflow, which minimizes the possibility of surface contamination. Targeted isolation from the water of geographically distant deep thermal aquifers in Chazhemto (Tomsk Region) and Belokurikha (Altay Region) resulted in the isolation of two new representatives of the class *Limnochordia*, described as *G. subterranea* gen. nov. sp. nov. and *C. subterranea* gen. nov. sp. nov. The deciphered metabolism of these two isolates suggests that *Geochordaceae* fam. nov. primarily live a heterotrophic life in the deep subsurface, respiring or fermenting simple sugars, fatty acids, and macromolecules derived from necromass or fossilized organic carbon from sedimentary rocks as electron donors.

*C. subterranea* L945^T^, unlike *G. subterranea* LN^T^, has limited ability to degrade polymers other than chitin and starch, but its metabolic flexibility is enhanced by the ability to grow lithoautotrophically on either H_2_ or CO. H_2_ is a common electron donor in the deep subsurface (Brazelton et al., 2012; Ruff et al., 2023). CO-based metabolism is well-recognized in hot springs and deep-sea volcanic vents (Robb and Techtmann, 2018), but the presence of carbon monoxide has also been postulated in subsurface environments with serpentinization under alkaline conditions (Brazelton et al., 2012). It is worth noting that the deep thermal waters of Belokurikha, from which the carboxydotrophic strain L945^T^ was isolated, have an alkaline reaction with a pH of 9.12. Chapelle and Bradley (2007) found that dissolved CO concentrations in shallow groundwater aquifers ranged from 0.0056 to 0.56 μg/L. It is also conceivable that the bacterium may use biogenic CO produced by sulfate-reducing and methanogenic prokaryotes, which are common inhabitants of the deep biosphere. For example, the sulfate-reducing *Desulfovibrio vulgaris* (recently reclassified as *Nitratidesulfovibrio vulgaris*) has been shown to produce a high amount of CO during the stationary growth phase (Voordouw, 2002). Thus, members of the *Geochordaceae* fam. nov. can switch from fermentation to anaerobic or aerobic respiration with organic or inorganic electron donors to optimize their performance under changing environmental conditions in the deep subsurface, where low energy fluxes are limited.

Of particular interest is the active aerobic respiration with O_2_ observed in both *Geochordaceae* strains. The co-existence of the strictly anaerobic sulfate-reducing *Thermodesulfovbrio* and aerobic *Meiothermus* was earlier demonstrated for reduced water from borehole 4E in Chazhemto (Lukina et al., 2023), which was used for the isolation of *C. subterranea* L945^T^, described in this study. Cultivation revealed both anaerobic sulfate reducers and heterotrophic aerobes in fracture water samples from the Thompson mine (Manitoba, Canada), collected approximately 1 km below the surface (Song et al., 2024). The heterogeneous conditions of the water-rock system in deep aquifers can result in special compartmentalization and the formation of toxic microzones. The traditional view of deep subterranean environments as devoid of oxygen and aerobic life (Onstott et al., 2019) has recently changed. Gaseous oxygen and oxygen-respiring prokaryotes have been found in deep terrestrial aquifers, which has been linked to the influx of oxygenated meteoric waters (An et al., 2013; Frank et al., 2016; Kadnikov et al., 2018a, 2019). Dissolved oxygen concentration reached 0.42–2.3 ml/L in fracture water from a deep gold mine in South Africa (Weinstein et al., 2019), from which the underground nematode *Halicephalobus mephisto* was isolated (Borgonie et al., 2011). Significant dissolved oxygen concentrations (0.52 ± 0.12 mg L^−1^) were recorded in geochemically mature groundwater samples collected from 138 groundwater monitoring wells in Alberta, Canada (Ruff et al., 2023). This amount of oxygen was suggested by the authors would be sufficient to support aerobic metabolisms in subsurface ecosystems on an unprecedented scale. *In-situ* production of molecular oxygen was confirmed by molecular oxygen (O_2_) isotope analysis and attributed to the microbial dismutation of chlorite and nitric oxide. In addition to the biological production of so-called “dark oxygen,” geochemical and geological processes like water radiolysis (Lin et al., 2005) and stress-induced generation via silicate mineral water interactions (He et al., 2021) may contribute to the appearance of oxygen in the deep subsurface. Thus, the growing evidence for aerobic metabolism, such as *Geochodaceae* fam. nov., should be considered when studying the deep biosphere.

The most active growth of both *Geochordaceae* strains in the laboratory cultures was observed when fumarate was used as an electron acceptor. Interestingly, there are reports of fumarate-respiring bacteria originating from the deep biosphere, despite the lack of clear evidence for the fumarate occurrence in these environments, including the strictly fumarate-respiring *Thermoanaerosceptrum fracticalcis*, isolated from an aquifer in fractured Paleozoic carbonate rocks at 863–923 meters below land surface in Nevada (Hamilton-Brehm et al., 2019). The only substrate supporting the growth of the bacterium was fumarate, whereas it was absent in the water sample used to isolate the strain. The source of fumarate in the subsurface remains unclear (Hamilton-Brehm et al., 2019 and references therein). Borehole drilling agents have been reported to be a source of fumarate (Borchardt, 1989). It is also conceivable that laboratory culture growth is not necessarily a model of bacterial metabolism in the environment. Bacteria can ferment organic substrates derived from decomposed fossil organics or necromass as hydrogen-scavenging community members reduce H_2_ pressure in the ecosystem. In the absence of an H_2_-scavenger, both *Geochordaceae* strains grew very slowly under fermentation conditions. Similarly, fumarate may promote optimal growth in the laboratory but does not affect the metabolism of the bacteriua in the environment.

Despite the fact that all cultivated members of the *Limnochordia* class have been isolated from water bodies, the majority of uncultivated members of the class in public databases originate from sludge and manure samples. A metagenome-based analysis of relative MAG abundance in digestors of a full-scale biogas plant revealed that *Limnochordia* was the most abundant MAG in all three digestors operating differently (Hassa et al., 2023). *Limnochordales* was the most frequent order revealed among MAGs binned from thermophilic composting cells at the São Paulo Zoo containing a mixture of lignocellulose and animal waste (Braga et al., 2021). The dominance of *Limnochrdia* phylotypes has been reported in sewage sludge (Shekhurdina et al., 2023) and digested cattle manure (Zhuravleva et al., 2022), where it was suggested that the MBA03 group of *Limnochordia* has syntrophic relationships with methanogenic archaea and is involved in direct interspecies electron transfer (DIET). This group also dominated the enrichment culture derived from microbial fuel cell cathodes inoculated with bovine and swine wastewater (Rago et al., 2018). The co-existence of the *Limnochordia* MBA03 group and *Methanosarcina* has been proposed to ensure DIET in full-scale methanogenic reactors (Calusinska et al., 2018). The transmission of electrical currents is known for the so-called cable bacteria, which are long filamentous multicellular members of the *Desulfobulbaceae* family (Cornelissen et al., 2018). The cell envelope of cable bacteria contains cell filaments that are continuous across the cell-to-cell junction. It is conceivable that the filamentous structures that we observed on the cell envelope of L945^T^ and LN^T^ strains (Figure 2) may be involved in electron transport and electroactivity of *Limnochordia* as occurs in cable bacteria.

Carboxydotrophic representatives of *Limnochordia* may constitute a notable share of the microbial community in ecosystems characterized by the presence of CO. An example of such an environment is the burning wastes of coal mining, where CO is the main gaseous product of incomplete combustion of coal (Karnachuk et al., 2023). MAG Bu05 belonging to *C. subterranea* was obtained from the coal-fire heated soil in Eastern Siberia (Kadnikov et al., 2023). Uncultivated members of the class *Limnochordia* were detected by 16S rRNA gene profiling in samples of burning lignite seams at Chagan-Uzun in the Altay Mountains during a long-term study (Kadnikov et al., 2018b; Karnachuk et al., 2023). Over a 6-year sampling period, its presence in the microbial community of heated rocks and soils of Chagan-Uzun ranged between 0.01% and 4.39%. *Limnochordales* phylotypes were discovered in heated rock samples from an abandoned coal mine near Kiselevsk, Kuzbass coal basin (Kadnikov et al., 2021a). The share of *Limnochordales* in the microbial community of thermal wastes from another Kuzbass coal mine ranged from 0.01% to 2.39% (Kadnikov et al., 2021b). CO is a common gas produced when organic waste and litter decompose, and it could be a potential substrate for carboxydothrophic *Geochordaceae*. CO concentrations of 120 μM were found in green waste compost and reached up to 10 mM in livestock waste composts (Hellebrand and Schade, 2008). In addition, carboxydotrophic bacteria have the potential to be used in biotechnological applications via syngas, a mixture of CO_2_, CO, and H_2_, produced as a byproduct of incomplete fossil fuel production (Alves et al., 2020).

Until now, *L. pilosa* was the only cultivated member of the class *Limnochordia* and the only option to verify the didermic (Gram-negative) character of the cell wall in this phylogenetic group. By screening 1,639 genomes of uncultivated *Firmicutes*, Taib et al. (2020) revealed outer membrane (OM) signatures in *Limnochordia*, strengthening their hypothesis of a didermic ancestor of *Firmicutes* and subsequent multiple OM losses in the phylum. The OM cluster of gene markers combined by Taib et al. (2020) was found in all 46 UBA *Limnochordia* genomes analyzed by the authors. A recent rooted bacterial phylogeny has confirmed the didermic character of the LBCA (Coleman et al., 2021). The OM of the cell wall of *G. subterreanea* sp. nov. and *C. subterreanea* sp. nov. clearly demonstrate the didermic (Gram-negative) character of the cell envelope of the novel cultivated representatives of the class *Limnochordia*.

Our study shows that careful consideration of genomic information may provide clues to obtaining laboratory cultures of yet-to-be-cultivated organisms. It is assumed that the lack of cultivated *Limnochordia* is due to overgrowth by other organotrophic bacteria in laboratory cultures when standard organic substrates are abundant. The discovery of less common metabolic pathways in the genome, such as the use of sorbitol in the case of *Geochorda subterranea* gen. nov. sp. nov., may result in the isolation of new microbes in the laboratory. Thus, genome availability may be an important prerequisite for cultivation. It is anticipated that novel *Limnochordia* isolates from sludge and agricultural waste, whose genomes are already available in UBA, will be cultured in the near future.

## Data Availability

The data presented in the study are deposited in the NCBI GenBank, accession numbers CP141614.1 (*Geochorda subterranea* LNT genome), CP141615.1 (*Carboxydochorda subterranea* L945T genome), PP660922.1 (16S rRNA gene of *Geochorda subterranea* LNT), and PP660921.1 (16S rRNA gene of *Carboxydochorda subterranea* L945T). The data can be found here: https://www.ncbi.nlm.nih.gov/nuccore/CP141614.1; https://www.ncbi.nlm.nih.gov/nuccore/CP141615.1; https://www.ncbi.nlm.nih.gov/nuccore/PP660922.1; https://www.ncbi.nlm.nih.gov/nuccore/PP660921.1.
