# Supplementary material for: Novel thermophilic genera Geochorda gen. nov. and Carboxydochorda gen. nov. from the deep terrestrial subsurface reveal the ecophysiological diversity in the class Limnochordia
Source: Front Microbiol. 2024 Sep 23;15:1441865. doi: 10.3389/fmicb.2024.1441865 (PMC11456536; doi:10.3389/fmicb.2024.1441865)
Supplement: Supplementary file 1 [file Data_Sheet_1.docx]

Supplementary Material

Novel thermophiles from deep terrestrial subsurface belonging to the genera *Geochorda* gen. nov. and *Carboxydochorda* gen. nov. shed light on the physiology and ecology of the class *Limnochordia*, represented mainly by uncultivated members

Olga V. Karnachuk ^1*^, Anastasia P. Lukina^1^, Marat R. Avakyan^1^, Vitaly V. Kadnikov^2^, Shahjahon Begmatov^2^, Alexey V. Beletsky^2^, Ksenia G. Vlasova^1^, Andrei A. Novikov^3^, Viktoria A. Shcherbakova^4^, Andrey V. Mardanov^2^ and Nikolai V. Ravin^2^

*** Correspondence:**Olga V. Karnachuk
[olga.karnachuk@green.tsu.ru](mailto:olga.karnachuk@green.tsu.ru)

## Supplementary Figure


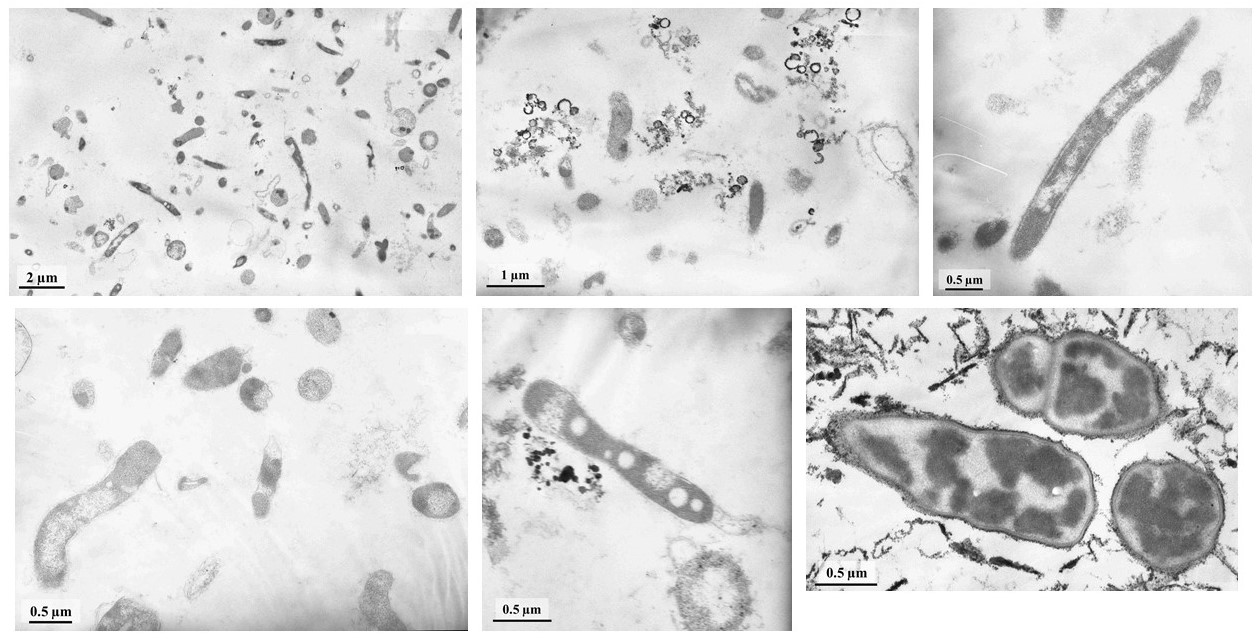


**Supplementary Figure 1.** TEM micrographs of ultrathin layers of the enrichment culture from borehole 5-P showing cells of different morphotypes.

## Supplementary Table

Table S1 – Cellular fatty acids of strains LN^T^ and L945^T^ (%)

| **Name** | **ECL (CP-Sil 88)*** | **Strain L945** | **Strain LN** |
| --- | --- | --- | --- |
| Butanedioic | 12.000 | tr** | 0.7 |
| i-C13:0 | 12.553 | tr | ND*** |
| ai-C13:0 | 12.720 | tr | ND |
| i-C14:0 | 13.525 | 3.6 | 0.5 |
| C14:0 | 14.000 | 0.8 | 0.7 |
| i-C15:0 | 14.545 | 28.3 | 23.1 |
| ai-C15:0 | 14.732 | 46.1 | 15.0 |
| C15:0 | 15.000 | tr | tr |
| i-C16:0 | 15.535 | 7.9 | 16.3 |
| C16:0 | 16.000 | 2.5 | 13.6 |
| i-C17:0 | 16.542 | 3.2 | 7.7 |
| ai-C17:0 | 16.732 | 4.5 | 18.4 |
| C17:0 | 17.000 | ND | tr |
| i-C18:0 | 17.538 | tr | tr |
| C18:0 | 18.000 | tr | 2.0 |
| C18:1 ω9c | 18.513 | tr | 1.1 |
| 2OH-C14:0 | 18.956 | tr | tr |
| C18:2 ω6,9c | 19.480 | ND | tr |
| 2OH-C15:0 | 19.984 | tr | ND |
| 3OH-C14:0 | 20.908 | tr | ND |
| 2OH-C16:0 | 21.139 | tr | ND |
| 3OH-C15:0 | 21.804 | tr | ND |
| 2OH-C17:0 | 22.644 | tr | ND |
| 3OH-C16:0 | 22.859 | tr | ND |

*ECL, equivalent chain length for the Agilent CP-Sil 88 column (highly polar cyanopropyl phase)

**tr, traces (<0.5%)

***ND, not detected

| **Table S2. Main characteristics of genomes used for comparative analysis with DRAM tool** | | | | |  |  |  |
| --- | --- | --- | --- | --- | --- | --- | --- |
|  |  |  |  |  |  |  |  |
| **Assembly** | **Name** | **Completeness (%)** | **Contamination (%)** | **Contigs** | **Contig N50 (bp)** | **Assembly size (bp)** | **GC Content** |
| GCA_035593265.1 | strain LN | 96.99 | 0.51 | 1 | 3019339 | 3019339 | 0.71 |
| GCA_035593305.1 | strain L945 | 99.07 | 0.46 | 1 | 3313549 | 3313549 | 0.69 |
| GCA_014896295.1 | MAG Bu05 | 97.26 | 1.81 | 8 | 492192 | 2886589 | 0.7 |
| GCA_014896305.1 |  | 91.97 | 0.15 | 58 | 176092 | 2558240 | 0.63 |
| GCA_014896315.1 |  | 96.78 | 0.18 | 20 | 285999 | 2594305 | 0.61 |
| GCA_012839805.1 |  | 96.4 | 0.93 | 51 | 83401 | 2782747 | 0.64 |
| GCA_001544015.1 | strain L. pilosa | 99.95 | 0.89 | 1 | 3817036 | 3817036 | 0.7 |
| GCA_017577755.1 |  | 86.93 | 0.45 | 60 | 65229 | 2203544 | 0.7 |
| GCA_017577405.1 |  | 86.34 | 0.27 | 52 | 86482 | 2250207 | 0.7 |
| GCA_012799445.1 |  | 81.18 | 1.31 | 450 | 6053 | 2153744 | 0.69 |
| GCA_017577945.1 |  | 97.3 | 0.88 | 19 | 360718 | 1960106 | 0.67 |
| GCA_003387805.1 |  | 91.34 | 3.4 | 454 | 6687 | 2368537 | 0.69 |
| GCA_017656145.1 |  | 98.95 | 0.48 | 116 | 46178 | 2571400 | 0.65 |
| GCA_002159155.1 |  | 95.84 | 1.1 | 304 | 12917 | 2774856 | 0.66 |
| GCA_017578085.1 |  | 95.24 | 2.07 | 236 | 13077 | 2335142 | 0.67 |
